# Elevated Serum C-Reactive Protein and Markers of Sleep Disordered Breathing

**DOI:** 10.1155/2012/914593

**Published:** 2012-02-19

**Authors:** R. Constance Wiener, Rouxin Zhang, Anoop Shankar

**Affiliations:** ^1^Department of Dental Practice and Rural Health, West Virginia University, P.O. Box 9448, Health Sciences North, Morgantown, WV 26506, USA; ^2^Department of Community Medicine, West Virginia University, P.O. Box 9190, Health Sciences North, Morgantown, WV 26506, USA

## Abstract

*Background*. Previous studies indicated sleep-disordered breathing (SDB) is associated with cardiovascular disease (CVD). Systemic inflammation is recognized as a risk factor for CVD. Studies examining SDB and inflammation are limited. *Methods*. We studied sleep duration, snoring, snorting, and daytime sleepiness, and an additive SDB score. The main outcome was a C-reactive protein (CRP) of >1 mg/dL. *Results*. Snoring, snorting, daytime sleepiness, and sleeping >7 or <7 hours, and the additive score were significantly associated with high CRP. The additive score was not associated in men but moderately associated in women in a multivariable model adjusting for age, gender, race/ethnicity, education, smoking, hypertension, alcohol intake, physical activity, body mass index, depression, diabetes, hypertension, and total cholesterol (*P*-interaction  = 0.42). For race/ethnicity, the association was strongest in Mexican Americans/others, modest in Non-Hispanic whites, and absent in Non-Hispanic blacks (*P*-interaction  = 0.07). *Conclusions*. The association between SDB and high CRP was present mainly in women and Mexican Americans, implying SDB has a residual, independent association with inflammation after controlling for lifestyle and metabolic risk factors like BMI, physical activity, depression, diabetes, and cholesterol.

## 1. Introduction

Sleep-disordered breathing (SDB) is a common, chronic disorder in which there is sleep-related repeated upper airway collapse resulting in episodes of hypoxemia and arousal [1]. Nationwide, SDB affects approximately 12–18 million people, nearly 4% of the population [2]. SDB is described as markedly undiagnosed [3] (especially in women [4]). SDB is a public health concern as it has been associated with cardiovascular disease (CVD) [5], motor vehicle crashes, [3, 6, 7] neuropsychological deficits [8], and mortality [9]. 

 Recent US and Japanese studies have shown an association between SDB markers and C-reactive protein (CRP), a biomarker of inflammation and predictor of future risk of CVD [10–13]. For example, strong positive associations occurred in a study of 69 healthy men, controlling for age, BMI, waist circumference, and percent body fat [1]. Also, a study of 18 men and 4 women showed elevated CRP levels with newly diagnosed obstructive sleep apnea in a multivariate analysis controlling for age, sex, BMI, smoking, alcohol, LDL, and HDL [10]. A study of 30 men with newly diagnosed obstructive sleep apnea and 14 obese controls showed higher CRP in those with obstructive sleep apnea than the obese controls. However, the studies were small in scope [10–13]. A larger study of 316 Japanese men showed SDB and CRP to be associated, however it trended to a higher association in those who were nonoverweight [14]. Also, not all studies have shown an association between SDB and CRP. The Wisconsin Sleep Cohort study of 907 adults aged 30–60, including 407 women, indicated that a significant association of CRP and SDB was not significant after adjusting for age, sex, and BMI [15].

 Additionally, studies examining the association between SDB and inflammatory markers by gender and race/ethnicity are limited and have conflicting results. In a study of 239 participants, including 83 women, where the statistical analysis was stratified by gender, the results indicated that SDB indices did not contribute to high CRP for men [16]. However, a study of 49 participants, including 12 women, showed that SDB markers were independently associated with high CRP [17]. Therefore, the role of gender in the association of self-reported SDB and CRP requires clarification. In a similar manner, although non-Hispanic Blacks and Hispanics have a disproportion of sleep disturbances [18], no examination of the markers of snoring, snorting, daytime sleepiness, and sleep duration and CRP was available in the literature. Therefore, we examined the association of markers of self-reported SDB and CRP stratified by gender and race/ethnicity in a large, nationally representative sample of US adults, after controlling for the main confounding factors.

## 2. Methods

 This study utilized data from the National Health and Nutrition Examination Survey (NHANES) 2005-06 and 2007-08. The study design and study methodology of the NHANES are available in detail elsewhere [19, 20]. Summarily, the NHANES is an on-going program of surveys assessing the health and nutrition of adults and children in the U.S by way of a stratified, multistage, probability sample representing the US civilian, noninstitutionalized population [19]. The survey uses interviews, physical examinations, and laboratory measurements of participants selected by counties, blocks, households, and individuals in the household with oversampling of non-Hispanic Blacks and Mexican Americans [19]. 

 CRP data was obtained for individuals aged twenty and above; therefore our study was restricted to those participants. Out of 20,497 participants in NAHNES survey, there were 10,914 subjects who were ≥20 years of age. We further excluded participants who were pregnant (*n* = 393), had prevalent CVD (*n* = 1,275), had missing information on SDB variables (*n* = 2,107), CRP, and other covariates (*n* = 651) included in the multivariable model. This resulted in 6,488 participants included in the final analysis.

### 2.1. Outcome of Interest: High CRP

High CRP level was the outcome of interest. High CRP was defined as >1 mg/dL, consistent with American Heart Association/Centers for Disease Control & Prevention (AHA/CDC) guidelines for identifying subjects with moderate/high risk of CVD [21]. Serum CRP was measured using latex-enhanced nephelometry. Details of the laboratory collection, processing, and analysis are available in the laboratory procedures manual [19, 20]. 

### 2.2. Assessment of Exposure: SDB

 The NHANES included a series of interview questions regarding sleep habits which were used to assess SDB. The questions included: “How much sleep do you usually get at night and on weekdays or workdays?”; “In the past 12 months, how often did you snore while you were sleeping?”; “In the past 12 months, how often did you snort, gasp, or stop breathing while you were asleep?”; “In the past month, how often did you feel excessively or overly sleepy during the day?” We developed four sleep variables from the questions: sleep duration; snoring; snorting; daytime sleepiness. Our categories for sleep duration were coded in hours into ≤5, 6, 7, 8, and ≥9; sleepiness, snorting, and snoring into “never or rare,” “sometimes” (2–4 times/week), and “often” or “almost always” (5 or more times/week).

 We also developed an additive summary SDB clustering score which was a composite of the four sleep variables after dichotomizing the variables utilizing clinical significance and previous literature [22–24]. In creating the four dichotomous SDB variables, a score of 1 was assigned if there was a report of sleep duration of five hours or less, snoring at least 3-4 nights/week, snorting at least 3-4 nights/week, and having daytime sleepiness of at least 5 times/month, and zero otherwise. These individual scores were then added to create the summary SDB clustering score. Therefore the additive summary SDB clustering score had a range of 0–4. A score of 0 reflected no sleep disturbances. A score of 4 indicated the simultaneous presence of all 4 sleep disturbances. 

### 2.3. Assessment of Covariates 

 The NHANES home interview had questions concerning age, gender, race/ethnicity, smoking status, alcohol intake (in grams/day), educational level, physical activity, body mass index, depression, diabetes, and hypertension. The educational level categorization was less than high school graduation, high school graduation, and more than high school graduation. The definition used for never smoking was the smoking of less than 100 cigarettes during one's lifetime. Former smokers were defined as those who had a lifetime smoking history of 100 or more cigarettes and were no longer smoking. Current smokers were defined as those who had a lifetime smoking history of 100 or more cigarettes and continued to smoke. Current alcohol drinking was defined as the consumption of one or more alcoholic drinks in the previous 12 months. Moderate physical activity was defined as participating in moderate-intensity sports, fitness, or recreations which resulted in a small heart and breathing rate increase. These activities included at least 10 minutes of brisk walking, cycling, swimming, or golf per week. 

 The medical examination center (MEC) examination provided anthropometric, physical, and laboratory evaluations. Body mass index (BMI) was determined as weight in kilograms divided by height in meter squared. Depression was defined as feeling down, depressed, or hopeless within the previous two weeks with a duration of more than half of the days or nearly every day. Three blood pressure measurements were taken with a mercury sphygmomanometer. The mean results were used for the systolic and diastolic pressures. If the mean systolic blood pressure was 140 mm Hg or above or the diastolic blood pressure was 90 mm HG or above, or the participant reported taking blood-pressure reducing medications, he or she was considered to have hypertension. 

 Total serum cholesterol levels were determined enzymatically using the Roche Hitachi 717 in 2005, Roche Hitachi 717 and 912 in 2006, and the Roche Modular P chemistry analyzer in 2007-2008. The details of blood collection, processing, and analysis are available in the laboratory procedures manuals [19, 20]. 

### 2.4. Statistical Analysis

Characteristics of the study participants by high CRP (levels > 1 mg/dL) were determined utilizing the chi square test or analysis of variance, as appropriate. We evaluated the associations between sleep variables (sleep duration, snoring, snorting, daytime sleepiness, and the *ad hoc* additive SBD clustering summary score) and CRP with two multivariable models. The first model adjusted for age (years), gender (male, female), race/ethnicity (non-Hispanic whites, non-Hispanic blacks, Mexican Americans, others), education (below high school, high school, above high school), smoking (never smoker, former smoker, current smoker), hypertension (absent, present), and alcohol intake (absent, present). In the second model we added physical activity (moderate physical activity), body mass index (obese, nonobese), depression (absent, present), diabetes (absent, present), and total cholesterol (mg/dL). In these analyses, we used linear regression models when the continuous CRP level was the dependent variable and logistic regression models when the categorical high CRP (levels > 1 mg/dL) was the outcome variable. We performed tests for trend with the individual sleep variables and the summary score as an ordinal variable in the multivariable models. We examined the consistency of associations with subgroup analysis stratified by gender and race/ethnicity. The analyses were weighted to adjust for the unequal probabilities of selection, oversampling, and nonresponse by using SUDAAN (version 8.0; Research Triangle Institute, Research Triangle Park, NC) and SAS (version 9.1.; SAS Institute, Cary, NC) software.

## 3. Results

The baseline characteristics of the study population are presented in Table 1. The study population was primarily white (72.6%), educated (58.6% were educated above high school), middle aged (44.8 years), and current drinkers of alcohol (74.3%). Normal weight, overweight, and obesity were equally distributed. Approximately one-half (52.8%) were never smokers, and the remainder was equally distributed as former smokers (23.6%) and current smokers (23.6%). Depression was reported in 5.1% of the population, and diabetes was present in 8.9%. 

Table 2 presents the ORs for the association between markers of SDB and the presence of CRP levels > 1 mg/dL. For sleep duration, compared with people who slept 7 hours (referent category), longer and shorter durations of sleeping had higher odds of having CRP levels > 1 mg/dL in both age-sex-adjusted and multivariable-adjusted models, although the multiple covariates in the latter model attenuated the association. 

 Similarly, for snoring, compared with 0–2 nights of snoring per week (referent category), snoring more frequently was associated with higher odds of having CRP levels > 1 mg/dL. We observed that compared to the age-sex and the multivariable model 1, there was attenuation of the association in the multivariable model 2, which additionally adjusted for physical activity, body mass index, depression, diabetes, hypertension, and total cholesterol. 

 For snorting, compared with snorting 0–2 nights per week (referent category), snorting more frequently was associated with higher odds of having CRP levels > 1 mg/dL. Here, we observed that even though there was a positive association in the age-sex and the multivariable model 1, with additional adjustment for physical activity, body mass index, depression, diabetes, hypertension, and total cholesterol in the multivariable model 2, the association was substantially attenuated and no longer present. 

 For daytime sleepiness, compared with 0-1 occurrences of daytime sleepiness per month (referent category), daytime sleepiness was associated with higher odds of having CRP levels > 1 mg/dL. We observed a positive association in the age-sex, and the multivariate model 1, which attenuated in the multivariable model 2. 

 We also derived an additive summary SDB clustering score by dichotomizing the SDB variables and taking their sum, resulting in a score ranging from 0 (no or minimal markers of sleep disorder) to 4 (the co-occurrence of all of the markers of sleep disorder). For the SDB clustering score, compared with a score of 0 (referent category), a higher sleep clustering score was associated with higher odds of having CRP levels > 1 mg/dL. The positive association persisted in the age-sex model and multivariable model 1, but was attenuated in the multivariable model 2 (Table 2). 

 Subsequently, we examined the association between the summary SDB clustering score and the odds of having CRP levels > 1 mg/dL within subgroup analyses of gender (Table 3) and race-ethnicity (Table 4). For the subgroup analysis by gender (Table 3), we found that higher summary SDB score was only weakly associated in men but moderately associated in women in the multivariable model 2 (*P*-interaction = 0.42). For the subgroup analysis by race/ethnicity (Table 4), the magnitude of association appeared to be the strongest in Mexican Americans and others, modestly in Non-Hispanic whites, and absent in Non-Hispanic blacks (*P*-interaction = 0.07). 

## 4. Discussion

In a large multiethnic sample of US adults, we found that markers of SDB, including short sleep duration (<7 hours), long sleep duration (>7 hours), occasional or frequent snoring or snorting (>2 night/week), and daytime sleepiness (>1/month) had moderately strong positive associations with the presence of CRP levels > 1 mg/dL in an initial multivariable model adjusting for age, sex, race/ethnicity, education, smoking, alcohol intake, and physical activity. However, additional adjustment for potential mediating factors such as BMI, depression, diabetes, hypertension, and total cholesterol substantially attenuated this association. Similarly, a summary SDB clustering score which counted the co-occurrence of various SDB markers showed that, when compared to those without SDB markers, those with clustering of SDB markers were associated with higher odds of having CRP levels > 1 mg/dL in the initial multivariable model. Here also the observed association was attenuated with additional adjustment for physical activity, body mass index, depression, diabetes, hypertension, and total cholesterol. 

 In the subgroup analysis examining the association between SDB clustering score and high CRP by gender, there was only a weak association in men, but a moderate association in women. In the subgroup analysis by race/ethnicity, the association between SDB clustering score and high CRP was strongly present in Mexican Americans, modestly present with a significant linear trend in Non-Hispanic Whites and absent in Non-Hispanic Blacks. 

 CRP is a nonspecific inflammatory biomarker that has been shown to be an important predictor of cardiovascular disease [14, 25, 26]. Additionally, CRP is implicated in inducting adhesion molecules, and the production of monocyte chemoattractant protein-1 and sensitizing endothelial cells to the cytotoxic process by CD4^+^T cells may also be an active atherosclerotic pathogenic agent, in and of itself [12].

 This study's main contribution to the existing literature concerning SDB is the demonstration in subgroup analyses that the SDB-CRP association is strong in Mexican Americans in the race/ethnicity subgroup analysis and that it is more strongly present in women in the gender subgroup analysis. The finding implies that SDB has a residual, independent association after the role of lifestyle and metabolic risk factors such as BMI, lack of physical activity, depression, diabetes, and total cholesterol in these subgroups. 

 The postulated mechanism of the association between SDB and CRP is complex. SDB may have the effects of hypoxemia, reoxygenation, hypercapnia, and arousals which activate systemic inflammation and the production of CRP [27]. 

 Similar studies have presented disparate results in analyzing the associations of CRP and SDB. Our study is consistent with a study of 69 men which indicated CRP is associated with SDB independent of adiposity [1]. Another study of 316 men also found an association of SDB and increased CRP, especially in men who were not overweight [15]. A third study of 935 women with type 2 diabetes also indicated SDB to be associated with increased CRP after adjusting for age, BMI, lifestyle, family history of diabetes, glycemic control, and medication use [28]. But a study utilizing the Wisconsin Sleep Cohort Study of 907 participants reported no association between CRP and SDB after controlling for age, sex, and BMI [14]. Utilizing the same study, but limiting the analysis to 589 women participants, SDB was 2.6 times more likely to occur postmenopausally than perimenopausally, after adjusting for age, body habitus, and certain lifestyle factors [29], indicating a potential hormonal influence associated with SDB. Our study will be largest to date that examined the association between SDB markers and CRP levels. We found that there was a modest positive association, which, in subsequent subgroup analysis, was found more strongly present in women and in Mexican Americans. However, there is a need to examine the gender-and race/ethnicity-specific associations between SDB and markers of inflammation in larger studies and employing alternative markers of inflammation such as interleukin-6 or tumor necrosis factor-alpha to prove or disprove our findings. 

 Additionally, research is needed to determine if SDB may be associated with chronic fatigue syndrome (CFS). CFS results in prolonged (6 months or greater) disabling daytime sleepiness and is associated with high levels of proinflammatory markers [30]. The mechanism of CFS appears to be an abnormal immunological response to an antigen/infection or a neuroendocrine problem resulting in a change in the CNS, particularly in young adults [30]. Examining the role of SDB may provide greater understanding of CFS. 

 This study's strengths include the large and national sample size of the study population and the breadth of the national survey which included factors which we were able to use as potential confounders. Our study limitations are the cross-sectional nature of the national study (which prohibits the making of causal inferences) and the self-reported nature of the SDB reports (which has the potential of nondifferential misclassification which would bias the association to the null). 

 In summary, this study shows that there is a positive association of SDB markers to high CRP in a national study of US adults, particularly for Mexican Americans and moderately for women. Prospective studies are needed to support the findings. These findings may be clinically useful in determining interventions to improve sleep and maintain lower levels of CRP.

## Figures and Tables

**Table 1:** Baseline characteristics of the study population.

Characteristics	Mean values ± standard error (SE) or percentages (number)
Total Number	6488
Women (%)	3158 (50.48%)
Age (years)	44.75 ± 0.45

Race/ethnicity (%)	
Non-Hispanic Whites	3211 (72.63%)
Non-Hispanic Blacks	1309 (10.04%)
Mexican Americans	1202 (7.94%)
Others	766 (9.40%)

Education categories (%)	
Below high school	1733 (16.87%)
High school	1576 (24.48%)
Above high school	3179 (58.64%)

Smoking (%)	
Never smoker	3453 (52.80%)
Former smoker	1539 (23.62%)
Current smoker	1496 (23.59%)

Alcohol intake, current drinker (%)	4444 (74.34%)
Moderate physical activity (%)	3017 (53.59%)

Body mass index (%)	
Normal weight	1970 (33.17%)
Overweight	2271 (33.89%)
Obese	2247 (32.94%)

Depression (%)	414 (5.12%)
Diabetes (%)	828 (8.92%)
Total cholesterol (mg/dL)	0.38 ± 0.01

**Table 2:** Association between sleep-disordered breathing variables and C-reactive protein levels > 1 mg/dL.

Sleep variables	Sample size (hypertension %)	Age, sex adjusted odds ratio (95% CI)	Multivariable-model 1* odds ratio (95% CI)	Multivariable-model 2^†^ odds ratio (95% CI)
Sleep duration (hours)				
≤5 hrs	120 (11.4%)	1.76 (1.23–2.53)	1.57 (1.11–2.23)	1.35 (0.93–1.97)
6 hrs	138 (8.1%)	1.18 (0.81–1.73)	1.13 (0.77–1.67)	1.11 (0.76–1.63)
7 hrs	141 (7.0%)	1 (referent)	1 (referent)	1 (referent)
8 hrs	157 (8.3%)	1.16 (0.87–1.54)	1.14 (0.85–1.52)	1.15 (0.84–1.58)
≥9 hrs	49 (10.9%)	1.50 (0.96–2.34)	1.44 (0.93–2.22)	1.61 (1.03–2.52)
*P*-trend		0.1275	0.3175	0.9488
Snoring (nights/week)				
0–2	247 (6.59%)	1 (referent)	1 (referent)	1 (referent)
3-4	130 (9.33%)	1.57 (1.20–2.05)	1.53 (1.17–2.01)	1.23 (0.93–1.62)
≥5	228 (0.34%)	1.98 (1.55–2.53)	1.90 (1.48 2.44)	1.26 (0.99–1.61)
* P*-trend		<0.0001	<0.0001	0.0619
Snorting (nights/week)				
0–2	509 (8.08%)	1 (referent)	1 (referent)	1 (referent)
3-4	44 (9.61%)	1.37 (0.93–2.03)	1.32 (0.89–1.95)	1.09 (0.73–1.64)
≥5	52 (11.94%)	1.72 (1.11–2.65)	1.64 (1.07–2.52)	1.06 (0.66–1.69)
* P*-trend		0.0054	0.0098	0.7288
Daytime sleepiness (times/month)				
0-1	323 (7.57%)	1 (referent)	1 (referent)	1 (referent)
2–4	154 (8.09%)	1.07 (0.85–1.36)	1.08 (0.84–1.38)	1.02 (0.78–1.33)
≥5	128 (11.54%)	1.53 (1.19–1.98)	1.47 (1.15–1.89)	1.27 (0.96–1.68)
* P*-trend		0.0026	0.0051	0.1289
Sleep summary score				
0	170 (6.13%)	1 (referent)	1 (referent)	1 (referent)
1	234 (8.45%)	1.52 (1.14–2.03)	1.470 (1.10–1.97)	1.16 (0.87–1.55)
2	148 (11.40%)	2.23 (1.68–2.95)	2.097 (1.57–2.80)	1.47 (1.07–2.02)
≥3	53 (13.46%)	2.67 (1.89–3.78)	2.400 (1.69–2.42)	1.40 (0.95–2.08)
* P*-trend		<0.0001	<0.0001	0.0228

*Model 1: adjusted for age (years), gender (male, female), race/ethnicity (non-Hispanic whites, non-Hispanic blacks, Mexican Americans, and others), education (below high school, high school, and above high school), smoking (never smoker, former smoker, and current smoker), and alcohol intake (absent, present).

^†^Model 2: adjusted for age (years), gender (male, female), race/ethnicity (non-Hispanic whites, non-Hispanic blacks, Mexican Americans, and others), education (below high school, high school, and above high school), smoking (never smoker, former smoker, and current smoker), alcohol intake (absent, present), physical activity (moderate physical activity), body mass index (obese, nonobese), depression (absent, present), diabetes (absent, present), and total cholesterol (mg/dL).

**Table 3:** Association between sleep variables and C-reactive protein levels > 1 mg/dL, by gender.

Sleep summary score	Sample size (hypertension %)	Age-adjusted odds ratio (95% CI)	Multivariable-model 1* odds ratio (95% CI)	Multivariable-model 2^†^ odds ratio (95% CI)
Men				
0	61 (4.30%)	1 (referent)	1 (referent)	1 (referent)
1	90 (5.49%)	1.27 (0.83–1.94)	1.20 (0.79–1.84)	1.12 (0.75–1.66)
2	62 (7.82%)	1.84 (1.14–2.97)	1.71 (1.04–2.81)	1.44 (0.89–2.34)
≥3	17 (7.42%)	1.80 (0.96–3.37)	1.56 (0.84–2.88)	1.24 (0.65–2.35)
*P*-trend		0.0084	0.0269	0.2038
Women				
0	109 (7.44%)	1 (referent)	1 (referent)	1 (referent)
1	144 (11.69%)	1.64 (1.10–2.46)	1.60 (1.07–2.39)	1.15 (0.77–1.72)
2	86 (16.31%)	2.42 (1.66–3.53)	2.29 (1.56–3.36)	1.49 (0.96–2.31)
≥3	36 (20.96%)	3.30 (2.10–5.17)	3.03 (1.91–4.80)	1.50 (1.02–2.21)
* P*-trend		<0.0001	<0.0001	0.0487

*Model 1: adjusted for age (years), race/ethnicity (non-Hispanic whites, non-Hispanic blacks, and Mexican Americans, others), education (below high school, high school, and above high school), smoking (never smoker, former smoker, and current smoker), and alcohol intake (absent, present).

^†^Model 2: adjusted for age (years), race/ethnicity (non-Hispanic whites, non-Hispanic blacks, and Mexican Americans, others), education (below high school, high school, above high school), smoking (never smoker, former smoker, current smoker), alcohol intake (absent, present), physical activity (moderate physical activity), body mass index (obese, nonobese), depression (absent, present), diabetes (absent, present), and total cholesterol (mg/dL).

**Table 4:** Association between sleep variables and C-reactive protein levels > 1 mg/dL, by race/ethnicity.

Sleep summary score	Sample Size (hypertension %)	Age-, sex-adjusted odds ratio (95% CI)	Multivariable-model 1* odds ratio (95% CI)	Multivariable-model 2^†^ odds ratio (95% CI)
Non-Hispanic Whites				
0	73 (5.55%)	1 (referent)	1 (referent)	1 (referent)
1	97 (7.87%)	1.59 (1.04–2.44)	1.54 (1.00–2.34)	1.23 (0.81–1.88)
2	71 (11.91%)	2.63 (1.80–3.85)	2.48 (1.66–3.68)	1.76 (1.14–2.72)
≥3	25 (14.09%)	3.24 (1.96–5.36)	2.79 (1.64–4.73)	1.62 (0.93–2.81)
*P*-trend		<0.0001	<0.0001	0.0173
Non-Hispanic Blacks				
0	48 (10.30%)	1 (referent)	1 (referent)	1 (referent)
1	62 (13.24%)	1.35 (0.82–2.22)	1.37 (0.82–2.28)	1.05 (0.61–1.80)
2	42 (14.02%)	1.52 (0.97–2.38)	1.55 (0.97–2.48)	0.96 (0.59–1.55)
≥3	10 (10.11%)	1.03 (0.42–2.49)	1.05 (0.43–2.56)	0.68 (0.29–1.60)
* P*-trend		0.2474	0.2032	0.4552
Mexican Americans and others				
0	32 (7.52%)	1 (referent)	1 (referent)	1 (referent)
1	53 (10.13%)	1.48 (1.00–2.18)	1.48 (0.98–2.22)	1.17 (0.75–1.83)
2	22 (10.42%)	1.58 (0.91–2.74)	1.53 (0.88–2.67)	1.17(0.64–2.13)
≥3	12 (24.46%)	4.40 (2.65–7.31)	4.21 (2.56–6.90)	2.96 (1.83–4.79)
* P*-trend	<0.0001	<0.0001	<0.0001	0.0047

*Model 1: adjusted for age (years), gender (male, female), education (below high school, high school, and above high school), smoking (never smoker, former smoker, and current smoker) and alcohol intake (absent, present).

^†^Model 2: adjusted for age (years), gender (male, female), education (below high school, high school, and above high school), smoking (never smoker, former smoker, and current smoker), alcohol intake (absent, present), physical activity (moderate physical activity), body mass index (obese, nonobese), depression (absent, present), diabetes (absent, present), and total cholesterol (mg/dL).
